# Genome-Wide Association Analysis Pinpoints Additional Major Genomic Regions Conferring Resistance to Soybean Cyst Nematode (*Heterodera glycines* Ichinohe)

**DOI:** 10.3389/fpls.2019.00401

**Published:** 2019-04-10

**Authors:** Dung T. Tran, Clinton J. Steketee, Jeffrey D. Boehm, James Noe, Zenglu Li

**Affiliations:** ^1^Institute of Plant Breeding, Genetics and Genomics and Department of Crop and Soil Sciences, University of Georgia, Athens, GA, United States; ^2^Department of Plant Pathology, University of Georgia, Athens, GA, United States

**Keywords:** soybean [*Glycine max* (L.) Merr.], soybean cyst nematode (*Heterodera glycines*) (SCN), resistance to *Heterodera glycines* (*Rhg*) genes, genome-wide association study (GWAS), quantitative trait loci (QTL), kompetitive allele specific polymerase chain reaction (KASP)

## Abstract

Soybean cyst nematode (*Heterodera glycines* Ichinohe) (SCN) is the most destructive pest affecting soybeans [*Glycine max* (L.) Merr.] in the U.S. To date, only two major SCN resistance alleles, *rhg1* and *Rhg4*, identified in PI 88788 (*rhg1*) and Peking (*rhg1/Rhg4*), residing on chromosomes (Chr) 18 and 8, respectively, have been widely used to develop SCN resistant cultivars in the U.S. Thus, some SCN populations have evolved to overcome the PI 88788 and Peking derived resistance, making it a priority for breeders to identify new alleles and sources of SCN resistance. Toward that end, 461 soybean accessions from various origins were screened using a greenhouse SCN bioassay and genotyped with Illumina SoySNP50K iSelect BeadChips and three KASP SNP markers developed at the *Rhg1* and *Rhg4* loci to perform a genome-wide association study (GWAS) and a haplotype analysis at the *Rhg1* and *Rhg4* loci. In total, 35,820 SNPs were used for GWAS, which identified 12 SNPs at four genomic regions on Chrs 7, 8, 10, and 18 that were significantly associated with SCN resistance (*P* < 0.001). Of those, three SNPs were located at *Rhg1* and *Rhg4*, and 24 predicted genes were found near the significant SNPs on Chrs 7 and 10. KASP SNP genotyping results of the 462 accessions at the *Rhg1* and *Rhg4* loci identified 30 that carried PI 88788-type resistance, 50 that carried Peking-type resistance, and 58 that carried neither the Peking-type nor the PI 88788-type resistance alleles, indicating they may possess novel SCN resistance alleles. By using two subsets of SNPs near the *Rhg1* and *Rhg4* loci obtained from SoySNP iSelect BeadChips, a haplotype analysis of 461 accessions grouped those 58 accessions differently from the accessions carrying Peking or PI 88788 derived resistance, thereby validating the genotyping results at *Rhg1* and *Rhg4*. The significant SNPs, candidate genes, and newly characterized SCN resistant accessions will be beneficial for the development of DNA markers to be used for marker-assisted breeding and developing soybean cultivars carrying novel sources of SCN resistance.

## Introduction

Soybean [*Glycine max* (L.) Merrill] is the most important economic crop in the legume family for both oil and food products. Currently, soybean is the second most grown crop in the U.S. behind only maize, totaling 33.8 million hectares planted annually, and grossing $40 billion in 2016 in the U.S. (ASA, 2018). However, soybean production in the U.S. is strongly undermined by SCN, a pest that caused yield losses of ∼3.4 million tons in 2014^1^. SCN was first reported in North Carolina, U.S. in 1954 (Riggs, 1975). Currently, SCN was found in every soybean producing state except West Virginia (Tylka and Marett, 2014; Wang et al., 2017).

Virulence of SCN is variable with 16 possible races determined by four selected differential lines, including PI 548402 (hereafter referred to as Peking), Pickett, PI 88788 and PI 90763 (Riggs and Schmitt, 1988). Of the 16 races, race 3 is considered the predominant race in the U.S. (Jackson, 2014). Later, due to increasing genotypic diversity of populations of SCN, the SCN population classification was modified by expanding to use seven indicator lines: Peking, PI 88788, PI 89772, PI 90763, PI 209332, PI 437654, and PI 548316. The new population classification system, termed HG Type, is determined by comparing SCN fecundity on each indicator line to the standard susceptible cultivar Lee 74 (Niblack et al., 2002).

Breeding SCN resistant cultivars and rotation with non-host crops are the most effective control methods for SCN (Kim et al., 2016). Screening soybean germplasm for resistance to SCN began as early as 1957 (Kim et al., 2016). Although many resistant cultivars have been reported, PI 88788 has been primarily used to breed resistant cultivars due to its desirable agronomic traits (Concibido et al., 2004). Use of a single source of SCN resistance such as PI 88788 or even multiple sources carrying the same SCN resistance gene may lead to a genetic shift in SCN populations by increasing the selection pressure. Several studies have reported that as many as 78% of SCN populations in Missouri and 8% of SCN populations in Illinois have already overcome Peking-type resistance (Mitchum et al., 2007; Niblack et al., 2008). The continued planting of soybean varieties with PI 88788 and Peking derived SCN resistance will only increase the number of SCN populations that have overcome their defense mechanisms. Therefore, it is paramount importance to soybean breeders to identify new SCN resistant germplasm sources from diverse genetic backgrounds that have different defense mechanisms than Peking and PI 88788.

The inheritance of SCN resistance is complex. More than 30 Quantitative Trait Loci (QTLs) controlling SCN resistance have been reported since 1994 by linkage mapping, with most of them being deemed only minor effect QTLs for SCN resistance (Concibido et al., 2004). Concibido et al. (1994) detected the first major effect SCN resistance QTL on Chr 18 [linkage group (LG) G] known as the *Rhg1* (Resistance to *H. glycines* 1) locus, reporting its presence in most of the resistant sources used for breeding commercial varieties, which included Peking, PI 88788, and PI 437654. At the *Rhg1* locus, allelic differences were detected between Peking and PI 88788, so the resistance alleles were denoted as *rhg1-a* (Peking type) and *rhg1-b* (PI 88788 type) (Kim et al., 2010). A 31.2 kb genomic segment of *rhg1-b*, with multiple copies causing phenotypic differences in SCN resistant lines was later identified (Cook et al., 2012). Within that 31.2 kb segment, three distinct genes contributing to SCN resistance were found within each repeat: *Glyma.18g02580*, *Glyma.18g02590*, and *Glyma.18g02610*. Of those genes, the *Glyma.18g02580* gene encodes a predicted amino acid transporter; the *Glyma.18g0290* gene encodes an α-SNAP protein, and the *Glyma.18g02610* gene encodes a protein with a wound-inducible protein 12 (WI12) (Cook et al., 2012). Chang et al. (1997) also reported a second major effect QTL providing SCN resistance, classified as the *Rhg4* locus on Chr 8 (LG A2), which was characterized as being present in some of the known resistant sources, such as Peking and PI 437654. One resistance gene that encodes a serine hydroxymethyltransferase (SHMT) was later found at the *Rhg4* region (Liu et al., 2012). Additional studies have discovered other QTLs for SCN resistance: *cqSCN-003* (*cqSCN* was designated by Soybean Genetics Committee^2^), located on Chr 16 from PI 88788 (Glover et al., 2004); *cqSCN-005*, located on Chr 17 (LG D2) from Hartwig (Kazi et al., 2010); *cqSCN-006* and *cqSCN-007*, on Chrs 15 (LG E) and 18 (LG G), respectively, from wild soybean accession PI 468916 (Kim and Diers, 2013), and finally *qSCN10*, located on Chr 10 (LG O) from PI 567516C (Vuong et al., 2010; Kadam et al., 2016).

Although QTL mapping in bi-parental populations is a powerful approach to identify genomic regions conveying SCN resistance, one shortcoming is that only genomic regions that have allelic variation between the two parents may be used to detect resistance. Moreover, a low amount of recombination in mapping populations may also decrease the mapping resolution. By contrast, genome-wide association studies (GWASs) utilize the genetic diversity of a panel of unrelated individuals to capture more recombination events by creating shorter linkage disequilibrium (LD) blocks, allowing for the identification of significant QTLs with higher resolution (Zhu et al., 2008). Furthermore, the development of high-throughput single nucleotide polymorphism (SNP) genotyping technology has supported the utilization of GWAS. In soybean, GWAS has been applied to dissect QTLs controlling seed quality, abiotic tolerance traits, disease resistance, and yield components (Jun et al., 2008; Wang et al., 2008; Bastien et al., 2014; Vaughn et al., 2014). GWAS has been previously used in several studies to locate genomic regions providing SCN resistance. Using a panel of 159 Chinese soybean accessions genotyped with 55 SSR loci, Li et al. (2011) identified three significant SSRs that were associated with SCN race 3 resistance on Chrs 7, 17, and 18. Bao et al. (2014) reported three QTLs on Chr 18 that condition HG Type 0 (race 3) resistance in a set of 282 soybean accessions from the University of Minnesota soybean breeding program that were genotyped with 1,536 SNP markers, which included *Rhg1, FGAM1*, and *Glyma.18g46201*. Han et al. (2015) assembled an association panel of 440 accessions that were genotyped with Specific Locus Amplified Fragment sequencing (SLAF-sequencing) and detected 12 SNPs that were significantly associated with HG Type 0 resistance and seven other SNPs that were significantly associated with HG Type 1.2.3.5.7 resistance. Of those 19 significant SNPs, eight were linked with the major SCN resistance QTLs *Rhg1* and *Rhg4*, whereas the remaining SNPs were distributed across 11 different chromosomes (Han et al., 2015). Additionally, Vuong et al. (2015) evaluated 553 soybean accessions in maturity groups (MGs) III to V and used SoySNP50K iSelect BeadChip genotyping data to detect 14 genomic regions associated with HG Type 0 resistance. Of these 14 genomic regions, three were associated with *Rhg1*, *Rhg4* and *qSCN-10* residing on Chrs 18, 8, and 10, respectively. For HG Type 2.5.7, Zhang et al. (2016) evaluated 235 wild soybean accessions genotyped with 52,041 SNPs using Illumina SoySNP50K iSelect BeadChip. They identified 10 significant SNPs on Chrs 18 and 19 associated with HG Type 2.5.7 resistance. Of these 10, four SNPs were located at known *rhg1* loci on Chr 18, and other SNPs on Chrs 18 and 19 were considered as novel regions. In 2017, another GWAS study for SCN race 1 with 1,032 wild soybean accessions with 42,000 SNPs was performed. Ten SNPs were reported as significantly associated with SCN race 1 resistance. Of them, three SNPs linked to known *rhg1*, two SNPs on Chr 4 and one SNP on Chr 18 were located at known QTLs (SCN 18-5; SCN 19-4; and SCN 37-2 respectively) while other SNPs located on Chrs 9 and 16 were considered as novel regions, explained 11.49–11.65% of phenotypic variation (Zhang et al., 2017).

The objectives of this study were to: (1) identify new germplasm sources of resistance to HG Type 0 (SCN race 3) by genotyping a diverse soybean germplasm panel consisting of 462 accessions with three KASP SNP markers developed at the *Rhg1* and *Rhg4* loci and screening those accessions with a greenhouse SCN bio-assay; (2) to use GWAS to pinpoint the genomic regions associated with HG Type 0 resistance among the 461 accessions by utilizing genotypic data from Illumina SoySNP50K iSelect BeadChips and phenotypic data from the greenhouse SCN bio-assay, and (3) to conduct a haplotype analysis of the 461 accessions near the *Rhg1* and *Rhg4* loci by using two subsets of SNPs on Chr 18 (103 SNPs) and Chr 8 (64 SNPs) obtained from the SoySNP50K iSelect BeadChips.

## Materials and Methods

### Plant Materials

A total of 448 accessions from MG 0 to VIII were selected from the United States Department of Agriculture (USDA) Soybean Germplasm Collection and the University of Georgia (UGA) soybean breeding program for SCN greenhouse screening procedures. The accessions were assembled from 28 different countries, with 62% of them originating from China, the center of origin for the domestication of soybean (Hymowitz, 1970). The accessions were selected based on the origin diversity. Of the assembled accessions, 289 accessions have not previously been phenotyped with SCN race 3, according to the U.S. National Plant Germplasm database. A total of 159 accessions with phenotypes available in Germplasm Resources Information Network (GRIN) database were also included in this panel to confirm their resistance and validate our KASP markers at *Rhg1* and *Rhg4* loci. The 448 accessions, along with six known SCN susceptible cultivars (Hutcheson, NC Roy, NC Raleigh, CNS, Lee, Lee 74) and one resistant line (G93-9009) were evaluated in the greenhouse. Additionally, the seven HG Type indicator lines (Peking, PI 88788, PI 90763, PI 437654, PI 209332, PI 89772, and PI 548316) were also included in phenotyping for HG Type determination.

### Greenhouse Phenotyping

Due to the large number of accessions and indicator lines (*n* = 462), the accessions were split into three sets for the greenhouse SCN bio-assay. Greenhouse assays were performed using SCN race 3 (HG Type 0) in the Plant Pathology Greenhouse at the University of Georgia, Athens, GA, United States in 2016. Plants were grown in cones (20.6 cm length and 4 cm diameter) that were filled with a steam-sterilized sandy loam soil. Cones were arranged into a randomized complete block design (RCBD) with four replications. Four seeds per accession were planted in each cone, which were thinned to a single seedling at 7–9 days after planting. Each seedling represented a single replicate, which was inoculated with a dispenser machine with 2,000 eggs placed in 3–4 mL of water. Cysts on the roots were counted approximately 40–60 days after inoculation when the number of cysts on Lee 74 (susceptible check) exceeded 50. Plant roots were individually washed free of soil, and then dried for 30 min. The female cysts were counted under a 20× lighted magnifying glass. The level of resistance was defined by the female index (FI) that was calculated based on the ratio between the mean numbers of cysts on a given line and on Lee 74, reported as a percentage (Niblack et al., 2002). The rating scale used to categorize SCN resistance was based on Schmitt and Shannon (1992): FI < 10% [Resistant (R)]; 10% < FI < 30% [Moderately resistant (MR)]; 30% < FI < 60% [Moderately Susceptible (MS)], and FI > 60% [(Susceptible (S)].

Based on the screening results of the three sets, 106 accessions which were rated as resistant or MR, were subsequently phenotyped with a new SCN race 3 population in the greenhouse during winter 2016. This new and more aggressive population of SCN race 3 (compared to HG type 0) was collected from Collins, Georgia in 2016. According to HG Type designation using the seven indicator lines this more aggressive SCN race 3 population was designated as HG Type 5. These accessions were sown in 10.2 cm wide clay pots filled with a fumigated sandy loam soil in December 2016. Pots were arranged in a RCBD with four replications. A heat mat was placed underneath the pots to maintain temperature at 28–30°C. Four seeds were planted in each pot, and then thinned to a single seedling per pot after 7–9 days.

### Target SNP Marker and 50K SNP Array Genotyping

Twelve young leaves per line were collected, and then freeze-dried for 48 h. DNA was extracted from soybean leaves using a modified CTAB method (Keim et al., 1988) and stored at -20° C until use. DNA concentration was quantified using a TECAN Infinite T1000 Pro (Tecan US, Inc., Morrisville, NC, United States) and diluted with water to 10–20 ng/μL for KASP assays.

All soybean accessions from the greenhouse screening were included for genotyping using KASP SNP markers at the *Rhg1* and *Rhg4* loci that were reported by Shi et al. (2015). KASP SNP marker GSM 381 was used for detecting the *rhg1* resistance allele at the *Rhg1* locus, and SNP marker GSM 383 was used to distinguish between the Peking (*rhg1-a*) and PI 88788 (*rhg1-b*) allele types at the *Rhg1* locus. SNP marker GSM 191 was used for identifying the resistance allele at *Rhg4* locus (Shi et al., 2015). The accessions were also genotyped with SNP marker GSM 039A on Chr 10 for southern root-knot nematode resistance (Pham et al., 2013). Genotyping was performed using the protocol reported by Pham et al. (2013). Briefly, KASP reactions were run in a 4 μL reaction, which included 2 μL of diluted DNA, 2 μl of KASP master mix, and 0.106 μl primer mix. The PCR fluorescent end reading was performed using a Light Cycler 480 Real Time PCR system (Roche, Germany).

More than 40,000 SNPs of the 461 accessions were obtained from the SoySNP50K Infinium Chip data^3^ and Soybean Breeding and Genetics Lab database at the UGA. SNPs were eliminated for analysis if they had no assigned physical position, greater than 20% missing data, or a minor allele frequency (MAF) less than 0.05. In total, 35,817 SNPs met these criteria and were used to conduct GWAS. The genotyping results of SNP markers GSM 381, GSM 383, and GSM 191 were also included in the GWAS analysis.

### Cluster Analysis Based on Haplotypes at the *Rhg1* and *Rhg4* Loci

Using SNPs obtained from the SoySNP50K Infinium Chip^3^ near *Rhg1* on Chr 18 (*n* = 103) and *Rhg4* on Chr 8 (*n* = 64), two neighbor-joining trees based on genetic distances at each of the *Rhg1* and *Rhg4* regions were constructed using TASSEL software (Bradbury et al., 2007). This software calculates genetic distance between each genotype using a modified Euclidean distance, where homozygote is 100% like itself and 50% similar if heterozygote (Bradbury et al., 2007). Then, the neighbor joining algorithm was used to create phylogenetic trees, and these results were visualized using Figtree software (Rambaut and Drummond, 2009). Because PI 670017 does not have SoySNP50K data cluster analysis was performed for 461 accessions.

*Rhg1* and *Rhg4* are two major effect loci that provide resistance to SCN race 3, as described above. At the *Rhg1* locus on Chr 18, three genes are known to contribute to SCN resistance (*Glyma.18g022400*, *Glyma.18g022500*, and *Glyma.18g022700* (corresponding to *Glyma18g02580*; *Glyma18g02590*, and *Glyma18g02610*, respectively, based on version 1.0 of Williams 82) (Cook et al., 2012). Based on the soybean reference genome sequence version 2.0 of Williams 82, approximately 500 kb flanking both sides of these three genes was selected for analysis. The selected 990-kb region, which included the *Rhg1* locus at Chr 18, resulted in the selection of 103 SNPs for the haplotype analysis.

Likewise, at the *Rhg4* locus on Chr 8, the *serine hydroxymethylytransferase* (*SHMT*) gene was attributed with conveying SCN resistance (Liu et al., 2012), as described previously. Two SNPs (ss715602757 and ss715602764) are situated close to this gene, thus a 0.5 Mb region (based on the soybean reference genome sequence version 2.0 of Williams 82) flanking both sides of these SNPs were selected for analysis, which resulted in the selection of 64 SNPs for the haplotype analysis at the *Rhg4* locus.

### Genome-Wide Association Analysis

The phenotyping data from the cyst counts were subjected to analysis of variance (ANOVA). A split-plot analysis for a mixed linear model was applied with blocks and sets being treated as random effects, while accession was considered as a fixed effect. R packages^4^ and JMP software (SAS Institute, Cary, NC, United States) were used to conduct ANOVA. No transformation of phenotype data was conducted prior to performing the GWAS analysis.

The genetic diversity of the plant accessions (*n* = 461) with SoySNP50K data available was analyzed using a principal component analysis (PCA) in the GAPIT R package (Lipka et al., 2012) and neighbor-joining (NJ) tree using TASSEL software (Bradbury et al., 2007). The PCA plot was visualized with TIBCO Spotfire (TIBCO Software, Inc., Palo Alto, CA, United States). The phylogenetic trees from TASSEL software were visualized with Figtree software (Rambaut and Drummond, 2009). The LD analysis was estimated using squared allele frequency correlation for pairs of SNPs from TASSEL software (Bradbury et al., 2007).

The statistical analysis R package GAPIT (Lipka et al., 2012) was used to conduct GWAS. In GAPIT, a compressed mixed linear model (CMLM) (Zhang et al., 2010) was used and the first five principal components (PCs) and a kinship matrix were included in this model to control Type I errors (false positives). For the CMLM model, the equation used was: y = μ + Xα + Pβ + Zu + e, where y is the phenotypic value; μ is the grand mean; X is the matrix coefficient to the fixed marker effects α; P is the matrix coefficient related to fixed PC effects β, and Z is the matrix coefficient related to random group effect u received from the compression algorithm. The threshold of significant value of association was False Discovery Rate-adjusted *P*-value (*P* < 0.001). A quantile–quantile (Q–Q) plot was visualized to evaluate how the model accounted for population structure. In addition, the generalized linear model (GLM) in TASSEL (Bradbury et al., 2007), and the enriched CMLM (ECMLM) model in the GAPIT package (Lipka et al., 2012) were performed to compare results with CMLM model. Both CMLM and ECMLM account for population structure and kinship while GLM with PCAs control for population structure.

### Candidate Genes of SCN Resistance

Three significant SNPs: GSM 381, GSM 383, and GSM 191 (Shi et al., 2015) at the *Rhg1* and *Rhg4* loci, situated on Chrs 18 and 8, respectively, were not included for candidate gene prediction in this study because resistance genes at those two genomic regions were previously reported (Cook et al., 2012; Liu et al., 2012). Herein, we focus on genes co-locating with the detected significant SNPs on Chrs 7 and 10 (deemed QTL regions) for a haplotype analysis using Haploview software (Barret et al., 2005). Genes located within each haplotype block which included a significant SNP associated with SCN resistance were selected as possible candidate genes. If a given significant SNP resulting from the GWAS analysis did not locate within a haplotype block, then gene models located within a 62.5 kb genomic region upstream or downstream of that SNP were considered. The protein sequences encoded by the predicted genes were retrieved from the Williams 82 soybean reference genome on SoyBase^5^. Two criteria were used to predict candidate genes responsible for SCN resistance: (1) if a gene was implicated as a gene providing disease resistance for nematodes or other pathogens in previous studies, or (2) if genes were located at genomic regions where the peak SNPs were placed as a result of the GWAS analysis. The gene models without functional annotations or belonging to unknown functional families were excluded.

## Results

### Greenhouse Evaluation for SCN Resistance

The mean FI of 462 accessions was 49.7% with a range of 0–200%, showing a continuous distribution (Supplementary Figure S1). The coefficient of variation was from 0 to 200%, with an average of 77.4%. Of the 462 accessions evaluated, seven indicator lines, seven controls, and 90 accessions were rated as resistant (R), with a calculated FI less than 10%. Additionally, 56 accessions were rated as moderately resistant (MR) with a calculated FI between 10 and 30% and 170 accessions were rated as moderately susceptible (MS) with a calculated FI between 30 and 60%. Finally, 146 soybean accessions were rated as highly susceptible (S) with calculated FI greater than 60% (Table 1 and Supplementary Table S1). Of the 146 resistant and MR accessions, 82 accessions were from China, 31 were breeding lines or cultivars from the U.S., and 32 accessions were from six other countries.

**Table 1:** Summary of greenhouse soybean cyst nematode (*Heterodera glycines* Ichinohe) (SCN) bio-assay phenotyping results of 462 soybean accessions for SCN resistance.

Rating	Female index^a^ (%)	No. of accessions
Resistant	0–10	90
Moderately resistant	10–30	56
Moderately susceptible	30–60	170
Susceptible	>60	146


*Rating scale of SCN resistance is as follows: Female Index (FI) < 10% (Resistant); 10% < FI < 30% (Moderately Resistant); 30% < FI < 60% (Moderately Susceptible), and FI > 60% (Susceptible) (Schmitt and Shannon, 1992). ^*a*^The level of resistance was defined by the Female Index (FI) that was calculated based on the ratio between the mean numbers of cysts on a given line and Lee 74 (susceptible check), reported as a percentage (Niblack et al., 2002).*

### SNP Marker Genotyping

Based on the genotype results of KASP SNP marker GSM 381 at the *Rhg1* locus (G = resistance allele), 90 accessions were predicted to be resistant to SCN race 3. According to genotype calls for SNP marker GSM 383 at the *Rhg1* locus (G = Peking type; C = PI 88788 type), 54 of 90 accessions were grouped as carrying Peking-type resistance, whereas 36 accessions were grouped as carrying PI 88788-type resistance. Combined with the phenotyping results, 88 of 90 accessions were rated as resistant or MR using the FI index. Two accessions, PI 578376 and PI 398823 were rated as susceptible. We speculate that this discrepancy is due to PI 398823 belonging to the Peking group at *Rhg1* with low copy number and not carrying a resistance allele at *Rhg4*. Previous studies have demonstrated that without the *Rhg4* resistance allele, the Peking-type *rhg1* resistance might not function efficiently for SCN resistance (Cook et al., 2014; Jiao et al., 2015). PI 578376 had a calculated FI of 31.8%, which is very close to being classified as MR.

At the *Rhg4* locus, 59 soybean accessions carried resistance alleles based on GSM 191 (G = resistance allele) genotyping results but three of the soybean accessions were not resistant based upon greenhouse phenotyping tests. No resistance allele at the *Rhg1* locus was found in these three accessions based on genotyping results using the GSM 381 KASP marker. Interestingly, 58 soybean accessions classified as being resistant and MR from greenhouse screening results did not carry either the *rhg1* or the *Rhg4* resistance alleles, suggesting that they might contain novel alleles for SCN resistance (Table 2). A large number (*n* = 311) of accessions rated moderately susceptible and susceptible in the greenhouse screening assay (including the six susceptible checks), matched the expected genotyping results by carrying susceptible alleles at both the *Rhg1* and *Rhg4* loci.

**Table 2:** Summary of genotyping results at known major soybean cyst nematode (*Heterodera glycines* Ichinohe) (SCN) resistance loci *Rhg1* and *Rhg4* on chromosomes 18 and 8, respectively, and phenotyping results of a greenhouse SCN bio-assay of 462 soybean accessions that were screened for SCN race 3 resistance.

			GSM	GSM	GSM
	Reaction to	No. of	381	383	191
Resistance alleles	SCN race 3	lines	*rhg1^a^*	*rhg1*	*Rhg4*
*rhg1* (Peking type) + *Rhg4*	RES^b^	50	GG	GG	GG
*rhg1 only* (Peking type)	RES/SUS	4	GG	GG	CC
*rhg1* (PI 88788 type) + *Rhg4*	RES	6	GG	CC	GG
*rhg1 only* (PI 88788 type)	RES	30	GG	CC	CC
*Rhg4 only*	SUS	3	TT	CC/GG	GG
**Absence of *rhg1* and *Rhg4***	RES	58	TT	CC	CC
**Absence of *rhg1* and *Rhg4***	SUS	311	TT	CC	CC


*A genotype call of GG denotes the soybean accession carries the desired resistance allele. ^*a*^Soybean accessions (*n* = 462) were genotyped using KASP SNP markers GSM 381 and GSM 383 at the *Rhg1* locus and GSM 191 at the *Rhg4* locus following protocols described by Shi et al. (2015). ^*b*^Rating scale of SCN was based on Schmitt and Shannon (1992): FI < 10% (Resistant); 10% < FI < 30% (Moderately Resistant); 30% < FI < 60% (Moderately Susceptible), and FI > 60% (Susceptible).*

Furthermore, 462 soybean accessions were genotyped using a functional KASP SNP marker GSM 039A (Pham et al., 2013) for southern root-knot nematode (*Meloidogyne incognita* Kofoid and White) resistance, which is one of the most damaging pests in the Southern U.S. Genotyping revealed that 135 accessions carried a previously identified allele providing resistance to this nematode. Of those 135 accessions, 58 accessions were rated as resistant to SCN based on the greenhouse screening assay, and of those 58 accessions, 18 appear to be carrying novel genes or alleles conveying SCN resistance. These 58 accessions will be further subjected to a greenhouse phenotyping for southern root-knot nematode resistance.

### Haplotype Analysis at *Rhg1* and *Rhg4* Loci

**FIGURE 1:**
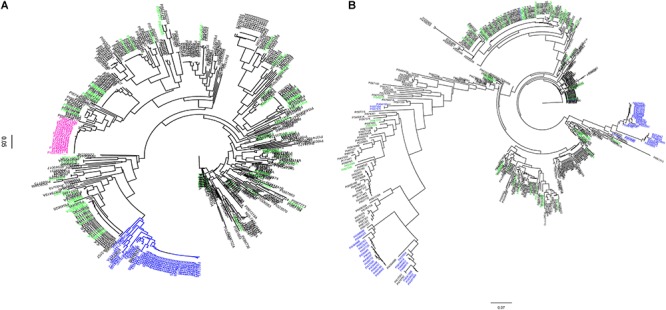
Dendogram of 461 soybean accessions generated using haplotype SNP markers at the *Rhg1* locus (*n* = 103) on chromosome (Chr) 18 and *Rhg4* locus (*n* = 64) on Chr 8. SNP data were obtained using the SoySNP50K iSelectBeadChips. **(A)** *Rhg1* locus: 990 kb region on Chr. 18. **(B)** *Rhg4* locus: a 997 kb on Chr 8. A total of 58 unique accessions (green) were grouped separately from PI 88788-type (pink) and Peking-type resistance (blue) at both of the *Rhg1* and *Rhg4* regions.

Using SNP data obtained from SoySNP50K Infinium Chips for these 461 soybean accessions, the cluster analysis based on 103 SNPs occupying a 990-kb region at the *Rhg1* locus on Chr 18 (LG G) separated soybean accessions carrying PI 88788-type (23 accessions) and Peking-type (50 accessions) alleles into two groups (Figure 1A). Two indicator lines, PI 209332 and PI 548316, located in the same group as PI 88788, whereas PI 437654, PI 89772, and PI 90783 were grouped with Peking, which is consistent with results from a previous study (Cook et al., 2012). Compared to the genotyping results generated from SNP markers (GSM 381 and GSM 383) at the *Rhg1* locus, 82% of the resistant accessions grouped into two clusters with Peking and PI 88788, respectively based on SoySNP50K data. A discrepancy was observed between our *rhg1* markers and the results of the haplotype analysis for 16 accessions.

Cluster analysis based on the other subset of SNPs (*n* = 64) occupying a 997-kb region at the *Rhg4* locus on Chr 8 placed 26 accessions into the Peking group, which was lower than the indicated genotyping results using KASP SNP marker GSM 191 at the *Rhg1* locus (59 accessions). Of these 59 accessions, 30 accessions were placed in one cluster group and three were placed in a different cluster group, which were distinct from the Peking cluster (Figure 1B). One explanation for the difference in results between the haplotype analysis and the KASP functional markers genotyping might be that no informative SNPs at the *Rhg1* and *Rhg4* loci were present in the SoySNP50K Chip data.

Interestingly, 58 accessions that were identified in greenhouse screening assays for SCN resistance and predicted not to carry resistance alleles using three functional KASP SNP markers (GSM 381, GSM 383, and GSM 191), were grouped separately from PI 88788 and Peking at both the *Rhg1* and *Rhg4* loci. Although there were three different clusters of soybean accessions having the *Rhg4*-resistance allele based on phylogenetic tree analysis at the *Rhg4* region, these 58 unique accessions were placed in different clusters (Figure 1A,B).

### LD Analysis and Population Structure

To understand the genetic architecture for SCN resistance and genetic relationship of 461 soybean accessions, more than 40,000 SNPs for these soybean accessions were retrieved from SoySNP50K Infinium BeedChip data^6^. In addition, three functional SCN KASP SNP markers GSM 381, GSM 383, and GSM 191 developed at the *Rhg1* and *Rhg4* loci were also included. After removing SNPs with MAF < 0.05 and missing data > 20%, a total of 35,817 SNPs were used for further analyses. The selected SNP markers per chromosome ranged from 1,301 on Chr 12 to 2,890 on Chr 18, with an average of 1,790 SNPs per chromosome. An average of one SNP per 19.7 kb was observed on Chr 13 and one SNP per 38.7 kb was observed on Chr 1, with an average of one SNP per 27.2 kb across all chromosomes. The recombination rate that impacts the resolution of association mapping is estimated by LD decay rates. The LD decay rate was measured by when the distance of the average pairwise correlation coefficient dropped to half of its maximum value. Herein, the LD decay distance was estimated to be about 125 kb and displayed a decreasing trend, suggesting that a high amount of recombination occurred among these soybean accessions (Supplementary Figure S2). LD decay distance was used to examine candidate genes within QTL regions and gene models that resided within a haplotype block or 125 kb genomic region of each significant SNP outside of haplotype block were considered.

**FIGURE 2:**
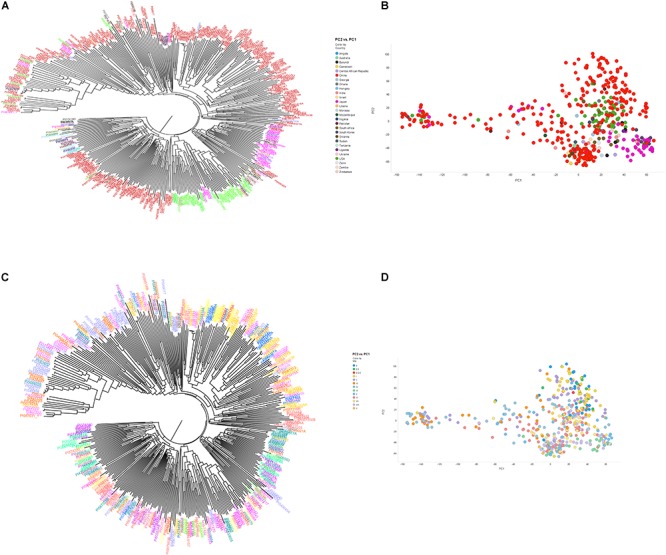
Neighbor-joining tree **(A,B)** and principal component analysis **(C,D)** depicting different clusters that were formed among 461 soybean accessions grouped by country of origin **(A,B)** and maturity group **(C,D)** (MG). **(A,B)** Clusters are colored according to country of origin (China: red; Japan: pink; United States: green; other colors: other countries). **(C,D)** Clusters are colored according to MG. The result of neighbor joining tree **(A,B)** was similar with PCA using first two components **(C,D)**.

The phylogenetic tree based on the 50K SNP data identified seven groups, which generally corresponded to the geographic origin of the accessions (Figure 2A). Most of the U.S. cultivars were placed in the same group, demonstrating that they were developed from limited ancestries. Two separate clusters were generated that represented accessions from Japan and South Korea. Due to a large number of accessions from China (62.1%), Chinese accessions were widely spaced. Accessions from other origins did not locate to unique clusters. These results indicate that place of origin and population structure were in general positively correlated (Figure 2A,B). Interestingly, 35 resistant and MR accessions including known resistant accessions (Peking, PI 88788, PI 090763, PI 437654, PI 089772, PI 438489B, PI 567516C, PI 087631-1, and PI 084751) belonged to one sub-cluster at whole-genome level. This result agreed with a previous GWAS study on SCN resistance (Vuong et al., 2015). In addition, length of time required for plant maturity as dictated by MG was included in the population structure analysis, and the accessions did not appear to be clustered by MG in the phylogenetic tree. PCA showed similar results to the neighbor joining tree-accessions test, revealing that the accessions did not tend to cluster based on MG (Figure 2C,D). Our results agree with a previous GWAS study, which indicated no correlation with population structure and MG (Zhang et al., 2016).

### GWAS for SCN Resistance

**FIGURE 3:**
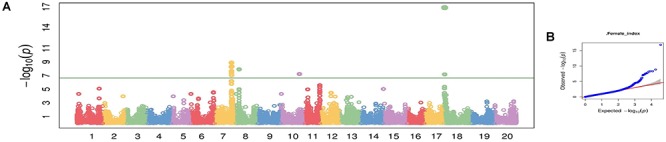
Manhattan plot **(A)** and quantile-quantile (Q-Q) plot **(B)** generated from genome-wide analysis for soybean cyst nematode (*Heterodera glycines* Ichinohe) (SCN) resistance among 461 soybean accessions using compressed mix linear model (CMLM) in GAPIT package.

Using CMLM in the GAPIT R package, the quantile–quantile plot visualized from the expected versus observed *P*-values from this model show that it adequately controlled population structure (Figure 3B). Using a threshold FDR *P*-value < 0.001, a total of 12 SNPs on Chrs 7, 8, 10, and 18 were found to be associated with HG Type 0 (race 3) SCN resistance using the GWAS analysis (Figure 3A and Table 3). The CMLM model successfully detected the KASP SNP marker GSM 381 at the *Rhg1* locus on Chr 18, and this marker had the highest peak (*P* = 1.24E-17) of all the markers tested. The other 11 SNPs identified using CMLM were as follows: eight SNPs on Chr 7, one SNP on Chr 8, one SNP on Chr 10, and one SNP on Chr 18. KASP SNP markers GSM 383 and GSM 191, located on Chrs 18 and 8 at known *Rhg1* and *Rhg4* regions, explained 34.1 and 26.2% of the phenotypic variation, respectively. Additionally, the genomic regions indicated by 9 SNPs on Chrs 7 and 10 were associated with HG Type 0 resistance. The eight significant SNPs identified on Chr 7 occupied a 0.5 Mb region located at 36.4–36.9 Mb, contributing 25–27% of phenotypic variation for SCN resistance. The genomic region for SCN resistance detected on Chr 7 overlapped with a previously recognized region (rs36423980: 36.4 Mb) associated with HG Type 2.5.7 (SCN race 1) resistance reported in another GWAS study (Webb et al., 1995; Zhao et al., 2017). The ss715606985 SNP on Chr 10 (resistance allele = GG) accounted for 25.6% of the phenotypic variation with an average FI index of 3.8%, which was significantly lower than the average FI index of the whole panel (49.76%). A total of 55 resistant and MR accessions identified using the greenhouse screening assay carried the ss715606985 resistance allele on Chr 10 based on GWAS. All these 55 accessions, including known resistant sources Peking, PI 89772, PI 437654, and PI 90763, carried resistance alleles at the *Rhg1* locus based on GSM 381 SNP marker allele calls. To date, no QTLs for SCN resistance at this region on Chr 10 have been reported using the populations derived from these known resistance sources.

**Table 3:** Summary of significant SNPs that were identified for soybean cyst nematode (*Heterodera glycines* Ichinohe) (SCN) resistance using multiple statistical GWAS models (GLM, CMLM, and ECMLM) among 462 soybean accessions phenotyped using a greenhouse SCN bio-assay for SCN resistance and genotyped using KASP SNP markers GSM 381 and GSM 383 at the *Rhg1* locus, KASP SNP marker GSM 191 at the *Rhg4* locus and SoySNP50K iSelect BeadChips.

							Avg FI for	Avg FI for
SNP ID^a^	Chr^b^	Position^c^	*P*-value	*R*^2^	R allele^d^	S allele^e^	the R allele	the S allele
		Bp					----------- % -----------	
ss715597408	7	36,368,238	4.42E-08	0.26	G	A	18.5	60.2
ss715597409	7	36,371,468	2.59E-08	0.26	G	A	18.3	60.5
ss715597410	7	36,376,909	2.23E-08	0.26	A	C	17.5	60.4
ss715597413	7	36,399,537	5.65E-09	0.26	G	T	18.0	60.5
ss715597431	7	36,449,014	1.38E-09	0.27	T	C	17.2	60.8
ss715597474	7	36,745,679	9.54E-08	0.25	A	G	28.3	61.5
ss715597497	7	36,907,461	4.59E-09	0.27	G	T	27.6	62.3
ss715597494	7	36,894,266	4.62E-09	0.27	T	C	29.4	62.3
GSM 191^f^	8	8,361,148	1.26E-08	0.26	G	C	4.6	56.2
ss715606985	10	40,672,699	6.15E-08	0.26	A	G	3.8	56.1
GSM 381	18	1,645,407	1.24E-17	0.34	G	T	4.2	60.7
GSM 383	18	1,643,660	6.81E-08	0.26	G	C	4.1	55.8


*^*a*^SNP ID derived from Illumina’s SoySNP50K iSelect Beadchips. ^*b*^Chromosome. ^*c*^SNP position according to the Williams 82 V2.0 reference genome in Soybase. ^*d*^Resistance allele. ^*e*^Susceptible allele. ^*f*^KASP SNP genotyping followed protocols described by Shi et al. (2015).*

### Candidate Genes and Ontologies for SCN Resistance

A total of 24 gene models were predicted using nine significant SNPs on Chrs 7 and 10 that were significantly associated with HG Type 0 resistance (Table 4). Based on haplotype analysis, six of the nine significant SNPs were located within three haplotype blocks (168, 109, and 6 kb) associated with SCN resistance on Chr 7. The three significant SNPs on Chr 7 and 10 did not fall within any haplotype blocks, but after scanning a 62.5 kb region flanking the significant SNPs, each were included in the 12 gene models. Based on information of gene ontology from SoyBase^7^, possible candidate genes were as follows: (1) a leucine rich repeat (LRR) protein kinase family protein gene, (2) a cytochrome P450 family protein gene, (3) a RING/U-box protein gene, (4) a DNA synthesis gene, (5) a transcription regulation gene and (6) others in miscellaneous groups. Of these listed candidate genes and their ontologies, some of them occupy domains on Chr 7 where R genes have previously been categorized, such as a (LRR) receptor gene (*Glyma.07g199500*), a cytochrome P450 gene (*Glyma.07g194400*), and a RING gene (*Glyma.07g196000*).

**Table 4:** Predicted genes for soybean cyst nematode (*Heterodera glycines* Ichinohe) (SCN) resistance located in three 100 kb haplotype block genomic regions near significant SNPs detected for SCN resistance on chromosomes 7 and 10 that were identified in a genome wide association study (GWAS).

Gene model	Chr^a^	Position	Function
		Bp	
Glyma.07g194200	7	36,276,988–36,282,713	DNA binding
Glyma.07g194400	7	36,294,553–36,297,818	Cytochrome P450 CYP2 subfamily
Glyma.07g194800	7	36,326,338–36,337,611	Protein binding
Glyma.07g195000	7	36,346,694–36,349,527	Intracellular protein transport
Glyma.07g195100	7	36,354,678–36,359,664	LRR-Protein kinase activity
Glyma.07g195300	7	36,383,490–36,386,028	Metabolic process
Glyma.07g195400	7	36,395,763–36,399,680	RING/ U-box superfamily protein
Glyma.07g195500	7	36,411,199–36,419,729	Nucleotide-excision repair
Glyma.07g195700	7	36,428,858–36,434,430	ATP binding∖mismatch repair
Glyma.07g195900	7	36,437,193–36,449,264	ATP binding
Glyma.07g196000	7	36,452,344–36,455,168	Protein binding
Glyma.07g196500	7	36,483,372–36,490,967	Ligase activity
Glyma.07g199000	7	36,759,783–36,761,919	DNA binding
Glyma.07g200100	7	36,873,150–36,873,836	Protein binding
Glyma.07g199500	7	36,809,934–36,815,777	LRR-like protein kinase
Glyma.07g199700	7	36,835,550–36,836,275	Regulation of transcription
Glyma.07g199900	7	36,854,378–36,857,292	Protein binding
Glyma.10g172700	10	40,646,539–40,649,573	Metabolic process
Glyma.10g172800	10	40,658,709–40,668,066	Protein import into mitochondrial outer membrane
Glyma.10g172900	10	40,666,359–40,670,242	Transmembrane transport
Glyma.10g173000	10	40,686,435–40,696,440	Protein kinase activity
Glyma.10g173100	10	40,699,391–40,701,312	Electron carrier activity
Glyma.10g173300	10	40,706,365–40,713,418	Protein binding
Glyma.10g173400	10	40,720,918–40,723,699	Plasma membrane


*Gene positions and functions are based on the Williams 82 version 2.0 reference genome in Soybase. ^*a*^Chromosome.*

## Discussion

### Identification of Potential Sources for SCN Resistance

Four hundred and sixty-two accessions were evaluated with HG Type 0 (SCN race 3) in this study. The FI of these accessions showed a large variation with a range of 0 to 200%. Of 159 accessions with phenotypes available in GRIN database, 15 accessions showed different reactions to SCN race 3. The difference could be due to the shifted nematode populations used for greenhouse nematode assays, different scales or susceptible checks used, escape or environmental effects (Supplementary Table S1).

To date soybean breeders have relied on just two primary resistant sources (Peking and PI 88788) to develop SCN resistant cultivars. In some cases, other SCN resistant lines have been identified, but they offer defense mechanisms that categorically fall into either Peking-type or PI88788-type resistance based on the major resistance QTL that they possess (Concibido et al., 2004). The Peking-type of SCN resistance requires the presence of resistance alleles at both the *Rhg1* and *Rhg4* loci, but PI 88788-type resistance requires only one preferable allele at *Rhg1* (Concibido et al., 2004). Due to limited genetic backgrounds of released SCN resistant cultivars, some SCN populations have overcome the PI 88788-type resistance (Niblack et al., 2008). In Georgia, a new aggressive SCN population was found that was designated as HG Type 5 based on HG Type test and race 3 based on the previous determination test with four indicator lines. Based on greenhouse phenotyping results with HG Type 0, 106 R and MR lines were phenotyped again with HG Type 5. Our study was able to identify 34 accessions which are resistant or MR to the aggressive HG Type 5.

Based on genes reported at *Rhg1* and *Rhg4* loci (Cook et al., 2012; Liu et al., 2012), three functional markers were designed to detect *rhg1* and *Rhg4* resistance alleles (Shi et al., 2015) to assist in identifying sources for SCN resistance other than *rhg1* and *Rhg4* alleles. The genotyping results of the three KASP SNP markers at the *Rhg1* and *Rhg4* loci indicated that 58 accessions rated resistant and MR from the greenhouse screening did not carry either the *rhg1* or the *Rhg4* resistance alleles. Haplotype analysis of these 58 accessions based upon SNPs at *Rhg1* and *Rhg4* regions also indicated that that these 58 accessions were not clustered with the accessions carrying the Peking-type or PI 88788-type haplotypes. Based on both the genotyping results of the KASP SNP markers at the *Rhg1* and *Rhg4* loci and haplotype analysis using SNPs surrounding the *Rhg1* and *Rhg4* genomic regions on Chrs 18 and 8, respectively, these 58 accessions may possess novel resistance alleles conferring SCN resistance that are different than Peking or PI 88788.

The origin for five of the 58 highest rated resistant accessions (PI 574484, PI 567403B, PI 561334, PI 603529, and PI 561329) was China, and each was also characterized as being highly resistant to aggressive SCN HG Type 5. Four of them (except PI 603529) have been characterized as having desirable agronomic and consumer traits, such as yellow seed coat, low lodging score (≤3 in scale 5), and low shattering (<2 in scale 5) (USDA-GRIN). Additionally, based on genotyping results using SNP marker GSM 039A, PI 561329 is also predicted to have resistance to southern root-knot nematode, as were 18 out of the 58 SCN resistant or MR accessions were identified. These 18 accessions appeared to be carrying novel genes or alleles conveying SCN resistance, as stated above. Therefore, these accessions could be valuable germplasm sources for resistance to two different nematode species: SCN (*Heterodera glycines* Ichinohe) and southern root-knot nematode (*Meloidogyne incognita* Kofoid and White).

### GWAS Analysis for SCN Resistance

To understand the genetic basis of SCN resistance in soybean, QTL mapping efforts have identified more than 30 QTLs on 17 of 20 soybean chromosomes in previous studies (Concibido et al., 2004; Guo et al., 2006; Kim et al., 2016). Among them, two major effect resistance QTLs on Chr 8 (*Rhg4*) and 18 (*Rhg1*) have been reported (Concibido et al., 2004). Recently, association mapping was conducted to explore the genetic architecture of soybean accessions to several traits including SCN resistance because of its advantages over linkage mapping. One of the advantages is increased mapping resolution (Korte and Farlow, 2013). However, one reported problem with using GWAS in their study was spurious association or Type 1 errors resulting from population structure and kinship because allele frequency differences were caused by different origins or MGs sharing the same ancestry (Korte and Farlow, 2013). In this GWAS study, a total of 461 soybean accessions from a wide range of MGs from 28 different countries were used. Based on population structure analysis, geographical diversification was slightly correlated, but no correlation was detected by MG. There are several models used in GWAS that include a PCA and kinship matrix to effectively control population stratification. However, depending on the statistical model used the results may differ, thus a Q–Q plot analysis is conducted to identify the model that best suits the data. In our analysis, multiple statistical models (GLM, CMLM, and ECMLM) were utilized to detect and verify genomic regions controlling SCN resistance (Supplementary Figure S3). Based on Q–Q plot results, all models were suitable for our analysis. Four significant SNPs (KASP SNP marker GSM 381 on Chr 18 and ss715597494, ss71559749, and ss715597431 on Chr 7) were identified using all GWAS models. The fact that these four SNPs were significant in each of the three statistical GWAS models tested indicates the importance of these SNPs for HG Type 0 resistance. Using GWAS, Vuong et al. (2015) detected two genomic regions on Chr 7 (36.5 and 43.0 Mb) associated with HG Type 0 (SCN race 3) resistance and Zhao et al. (2017) identified one locus on Chr 7 (36.4 Mb) associated with HG Type 2.5.7 (SCN race 1) resistance. Our detected region on Chr 7 overlapped with their recognized regions. The genomic regions might overlap with QTL regions identified on Chr 7 in PI 437654 (Webb et al., 1995). A recent publication also showed that the SNP marker, ss715597431 is associated with SCN resistance in soybean (Bayless et al., 2018). The authors concluded that the *N*-ethylmaleimide sensitive factor (NSF) gene which was located near SNP ss715597431 on Chr 7 might be co-inherited with the NSF gene at *Rhg1* locus on Chr 18. They found 855 soybean accessions from USDA germplasm which carried *rhg1* resistance allele are also homozygous for SNP ss715597431. Similarly, with our GWAS panel (461 accessions), we found 87 of 89 soybean accessions carrying resistance allele at *Rhg1* (both PI 88788 and Peking) had beneficial allele at ss715597431 locus. However, of these 87 accessions, two were rated as moderately and highly susceptible. PI 398823 carried Peking-type resistance allele at *Rhg1* locus, but it did not possess resistance allele at *Rhg4*. Additionally, five MR (FI: 10.7–28.9%) accessions that do not possess a resistance allele at *Rhg1* based on our genotyping results carry resistance allele at SNP ss715597431 locus. Further studies will need to be performed.

Additionally, two other QTLs on Chrs 8 and 10, respectively, were detected using two statistical models (GLM and CMLM) (Supplementary Figure S3). The genomic region on Chr 8 is associated with major resistance locus *Rhg4* (denoted by KASP SNP marker GSM 191). The genomic region at SNP ss715606985 on Chr 10 was located 1 Mb from the previously mapped QTL, *qSCN10*, identified from PI 567516C (Vuong et al., 2010, 2015). Additionally, ss715606985 was also reported for root-knot nematode and SCN race 3 and 5 resistance in a previous GWAS study (Chang et al., 2016). The genotyping results of SNP marker ss715606985 revealed that 55 of the resistant and MR accessions, including PI 567516C, carried the same resistance allele (A); interestingly, all of these 55 resistant accessions including Peking, PI 89772, PI 90763, and PI 437654, also carried the same resistance alleles as PI 567516C although no QTL controlling SCN resistance on Chr 10 was reported in bi-parental mapping populations derived from Peking, PI 89772, PI 90763, and PI 437654, as stated above. Furthermore, the results of the greenhouse screening indicated that the average FI of accessions carrying the resistance allele at SNP marker ss715606985 on Chr 10 was significantly low (3.8%), suggesting that this SNP marker could locate in a genomic region responsible for SCN resistance. This result highlights one of the advantages of using GWAS over bi-parental mapping populations by demonstrating that QTL mapping is limited by the number of recombination events occurring between two parents to map traits, whereas GWAS is able to capture more allelic diversity from a larger panel of accessions with a greater number of recombination events, and thus is able to significantly associate traits at genomic regions that bi-parental mapping populations are unable to detect.

The genomic regions containing SNPs with the FDR *P*-value < 0.05 were also examined to compare them with previous reported QTL. Two additional genomic regions on Chrs 6 (LG C2) and 11 (LG B1) were identified (Supplementary Table S2). The genomic region on Chr 6 (LG C2) flanked by two SNPs: ss715594958 and ss715594959 were overlapped with reported QTL on Chr 6 from PI 438489B (Yue et al., 2001). Thirty-seven resistant or MR accessions carried the same beneficial allele (A allele) at both SNPs. Of 37 accessions, 29 carried resistance allele at *Rhg1* based on KASP markers (18 accessions carried Peking allele and 11 carried PI 88788 allele).

On Chr 11 (LG B1), a total of eight SNPs with *P*-value < 0.05, which spanned a 144 kb region (position 32,834,795 to 32,978,048 bp) were identified (Supplementary Table S2). This region overlapped or located closely with previously reported QTL, which were identified from PI 438489B (27.7–32.4 Mb); PI 90763 (32.4–34.1 Mb); PI 404198A (33.6–34.1 Mb); and PI 437654 (29.7–32.2 Mb) (Yue et al., 2001; Guo et al., 2005, 2006; Wu et al., 2009). This region is also close to the regions identified in a previous GWAS study for SCN resistance by Li et al. (2016). Of these eight SNPs, ss715610417, ss715610420, and ss715610421 were located in the same haplotype block containing *Glyma.11g234500 (GmSNAP11*), which belonged to same SNAP family with *Glyma.18g02590 (GmSNAP18*), which is one of the *Rhg1* genes (Cook et al., 2012). The results from Lakhssassi et al. (2017) indicated that *GmSNAP18* and *GmSNAP11* shared 92.4% amino acid sequence similarity, *GmSNAP11* contributed to SCN resistance as an additive effect, and the FI of genotypes having both *GmSNAP18* and *GmSNAP11* was lower than that of genotypes containing only *GmSNAP18*. Following up this observation, we examined 66 accessions carrying resistance alleles at *Rhg1* locus which also carry three beneficial alleles of three closest SNPs to *GmSNAP11*. Of these 66 accessions, 65 were rated as resistant or MR to HG Type 0. One accession, PI 398823 was highly susceptible since it only carries the Peking type allele at *Rhg1* locus, not *Rhg4* allele.

### Candidate Genes for SCN Resistance

To date, the two primary candidate genes controlling SCN resistance at the *Rhg1* and *Rhg4* loci have been cloned. However, it is vital for soybean breeders to discover additional genes conferring SCN resistance in order to maintain and enhance resistance as well as have alternative solutions when *rhg1* and *Rhg4* lose effectiveness, which is already occurring with *rhg1* (Peking-type resistance). Based on a single marker or haplotype, GWAS has not only identified new significant genetic regions associated with SCN resistance, but also predicted new candidate resistance genes within these regions other than *Rhg1* and *Rhg4*. Due to the large number of gene models that occupy the haplotype blocks surrounding the significant SNPs that were identified using GWAS in this study, it was difficult to specify the candidate genes conferring SCN resistance. However, based on the gene ontologies in SoyBase for the genes near the significant SNPs identified using GWAS herein, we propose that 24 different gene models on Chrs 7 and 10, respectively, may play a role in providing SCN resistance. Of them, three belong to chromosomal domains with previously reported plant disease resistance (R) genes, such as LRR, cytochrome 450 and RING/U box (Han et al., 2015). Further research is needed to confirm if these genes might be responsible for conveying SCN resistance in soybean.

## Author Contributions

ZL conceived the project, provided oversight of the experiments, interpreted the results, and edited the manuscript. DT performed the experiments, carried out greenhouse phenotyping and population genotyping, performed data analysis, and drafted the manuscript for review. CS was involved in setting up the project, initial data analysis, and interpretation and editing manuscript. JB participated in the result interpretation and edited manuscript. JN provided oversight for greenhouse phenotyping. All authors read and approved the final manuscript.

## Conflict of Interest Statement

The authors declare that the research was conducted in the absence of any commercial or financial relationships that could be construed as a potential conflict of interest.

## Footnotes

1 http://extension.cropsciences.illinois.edu/fieldcrops/diseases/yield_reductions.php

2 http://soybase.org

3 http://soybase.org

4 http://cran.r-project.org

5 http://soybase.org

6 http://soybase.org

7 http://soybase.org

## Supplementary Material

Figure S1 Distribution of female index among 461 soybean accessions using Origin 6.0 software (OriginLab Corporation, Northampton, MA).

Figure S2 Genome-wide linkage disequilibrium (LD) decay plot for 461 soybean accessions based on 35,817 SNPs. Linkage disequilibrium, measured as r^2^ between pairs of marker was plotted against the genetic distance (cM). The fitting curve (blue line) illustrated the model fit to LD decay. The estimated LD decay rate was about 125kb which was measured by when the r^2^ dropped to half of its.

Figure S3 Manhattan plots and Q- Q plots generated from genome-wide analysis using TASSEL software and GAPIT package. **(A)** General linear model (GLM) generated by TASSEL; **(B)** Compressed mix linear model (CMLM); and **(C)** enriched compressed mix linear model (ECMLM) generated by GAPIT package. The -log10 P values from a genome-wide scan are plotted against the position on each of the 20 chromosomes. The dash lines indicated the genome-wide significant with red points represented significant SNPs. Right: Q-Q plot showing the expected P value compared to the observed value. The red line is X = Y line indicated the null hypothesis: no true association. Q-Q plot curved at the tail which implied the small number of true SNPs association.

Table S1 Soybean accessions and their phenotypes.

Table S2 Summary of 26 additional SNPs that were identified for SCN resistance using GWAS model (CMLM) among 461 soybean accessions. Threshold FDR value is *P*-value.
