# Supplementary figures and images for: Genome-Wide Association Analysis Pinpoints Additional Major Genomic Regions Conferring Resistance to Soybean Cyst Nematode (Heterodera glycines Ichinohe)
Source: Front Plant Sci. 2019 Apr 10;10:401. doi: 10.3389/fpls.2019.00401 (PMC6470319; doi:10.3389/fpls.2019.00401)

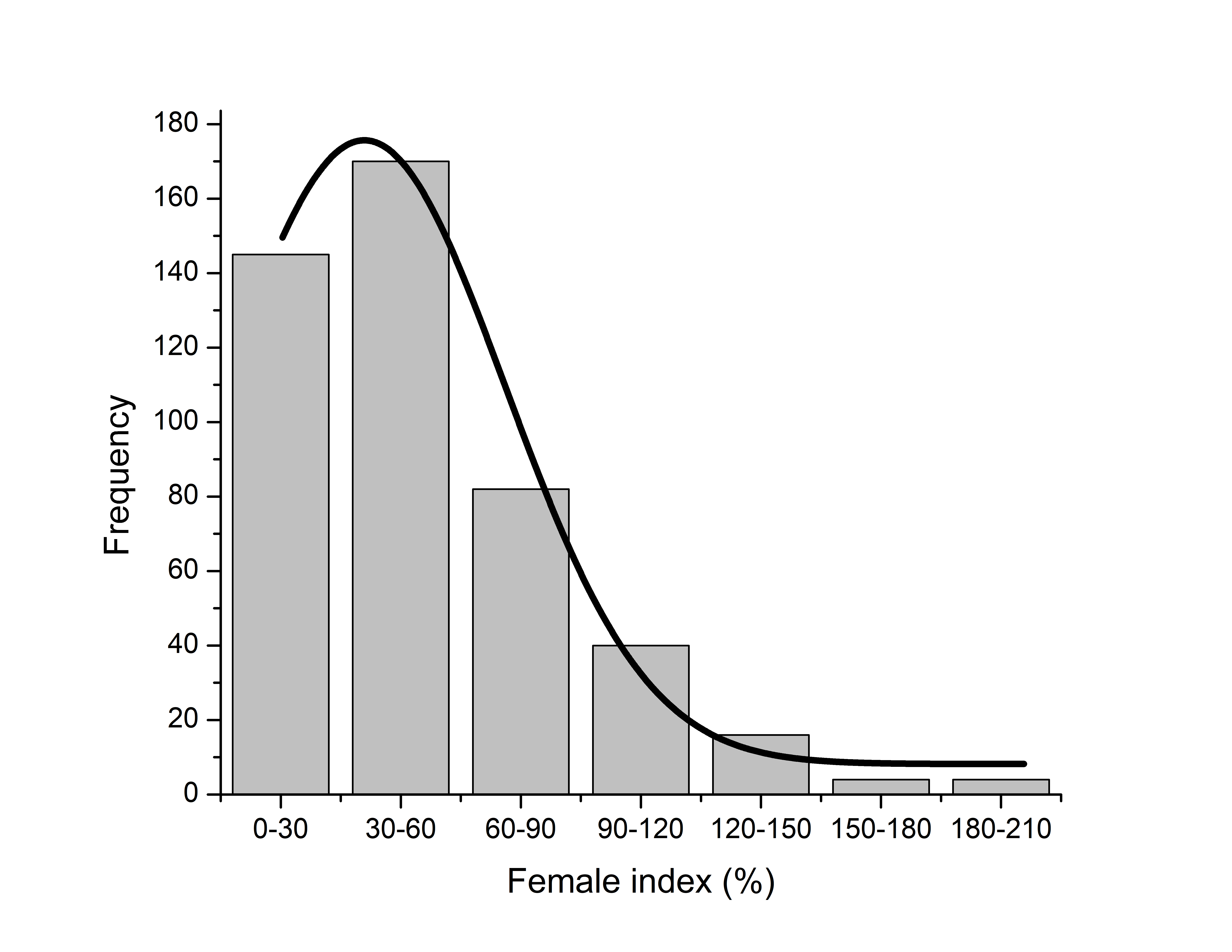

Supplement: Figure S1 — Distribution of female index among 461 soybean accessions using Origin 6.0 software (OriginLab Corporation, Northampton, MA). [file Image_1.JPEG]

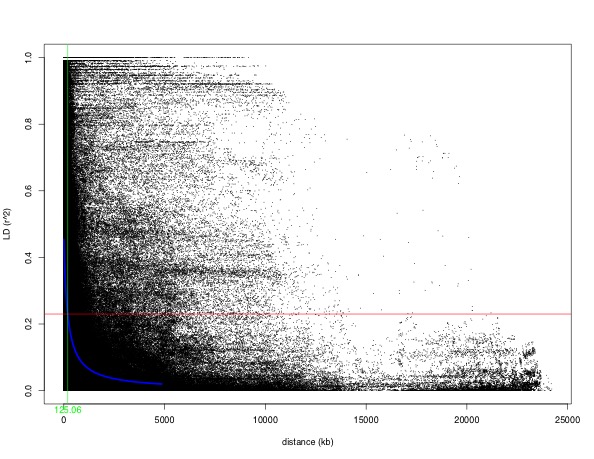

Supplement: Figure S2 — Genome-wide linkage disequilibrium (LD) decay plot for 461 soybean accessions based on 35,817 SNPs. Linkage disequilibrium, measured as r2 between pairs of marker was plotted against the genetic distance (cM). The fitting curve (blue line) illustrated the model fit to LD decay. The estimated LD decay rate was about 125kb which was measured by when the r2 dropped to half of its. [file Image_2.JPEG]

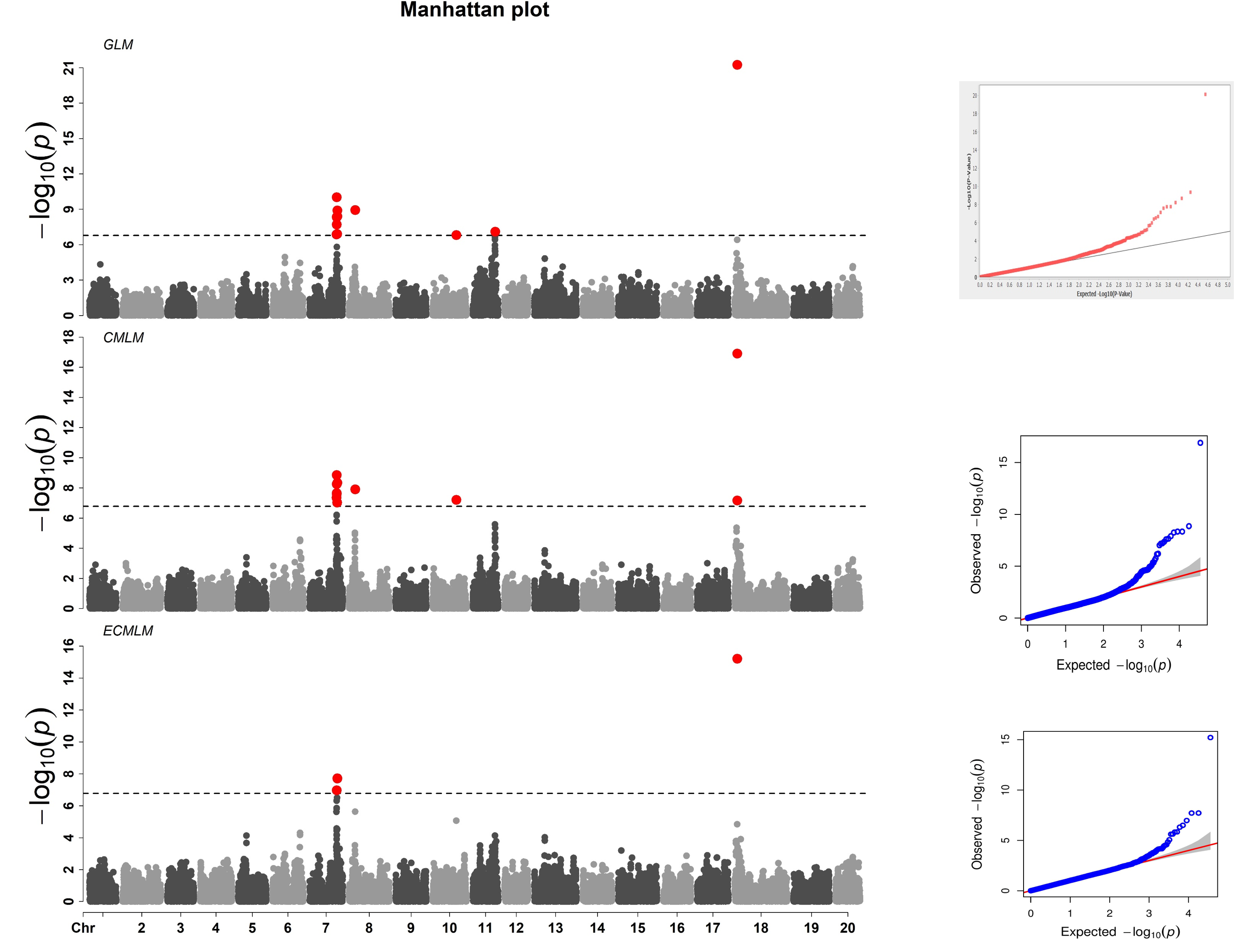

Supplement: Figure S3 — Manhattan plots and Q- Q plots generated from genome-wide analysis using TASSEL software and GAPIT package. (A) General linear model (GLM) generated by TASSEL; (B) Compressed mix linear model (CMLM); and (C) enriched compressed mix linear model (ECMLM) generated by GAPIT package. The -log10 P values from a genome-wide scan are plotted against the position on each of the 20 chromosomes. The dash lines indicated the genome-wide significant with red points represented significant SNPs. Right: Q-Q plot showing the expected P value compared to the observed value. The red line is X = Y line indicated the null hypothesis: no true association. Q-Q plot curved at the tail which implied the small number of true SNPs association. [file Image_3.PNG]
